# Model Combining Tumor Molecular and Clinicopathologic Risk Factors Predicts Sentinel Lymph Node Metastasis in Primary Cutaneous Melanoma

**DOI:** 10.1200/PO.19.00206

**Published:** 2020-04-14

**Authors:** Domenico Bellomo, Suzette M. Arias-Mejias, Chandru Ramana, Joel B. Heim, Enrica Quattrocchi, Sindhuja Sominidi-Damodaran, Alina G. Bridges, Julia S. Lehman, Tina J. Hieken, James W. Jakub, Mark R. Pittelkow, David J. DiCaudo, Barbara A. Pockaj, Jason C. Sluzevich, Mark A. Cappel, Sanjay P. Bagaria, Charles Perniciaro, Félicia J. Tjien-Fooh, Martin H. van Vliet, Jvalini Dwarkasing, Alexander Meves

**Affiliations:** ^1^ SkylineDx, Rotterdam, the Netherlands; ^2^ Mayo Clinic, Rochester, MN; ^3^ Mayo Clinic, Scottsdale, AZ; ^4^ Mayo Clinic, Jacksonville, FL; ^5^ Gulf Coast Dermatopathology Laboratory, Tampa, FL

## Abstract

**PURPOSE:**

More than 80% of patients who undergo sentinel lymph node (SLN) biopsy have no nodal metastasis. Here, we describe a model that combines clinicopathologic and molecular variables to identify patients with thin- and intermediate-thickness melanomas who may forgo the SLN biopsy procedure because of their low risk of nodal metastasis.

**PATIENTS AND METHODS:**

Genes with functional roles in melanoma metastasis were discovered by analysis of next-generation sequencing data and case-control studies. We then used polymerase chain reaction to quantify gene expression in diagnostic biopsy tissue across a prospectively designed archival cohort of 754 consecutive thin- and intermediate-thickness primary cutaneous melanomas. Outcome of interest was SLN biopsy metastasis within 90 days of melanoma diagnosis. A penalized maximum likelihood estimation algorithm was used to train logistic regression models in a repeated cross-validation scheme to predict the presence of SLN metastasis from molecular, clinical, and histologic variables.

**RESULTS:**

Expression of genes with roles in epithelial-to-mesenchymal transition (glia-derived nexin, growth differentiation factor 15, integrin-β3, interleukin 8, lysyl oxidase homolog 4, transforming growth factor-β receptor type 1, and tissue-type plasminogen activator) and melanosome function (melanoma antigen recognized by T cells 1) were associated with SLN metastasis. The predictive ability of a model that only considered clinicopathologic or gene expression variables was outperformed by a model that included molecular variables in combination with the clinicopathologic predictors Breslow thickness and patient age (area under the receiver operating characteristic curve, 0.82; 95% CI, 0.78 to 0.86; SLN biopsy reduction rate, 42%; negative predictive value, 96%).

**CONCLUSION:**

A combined model that included clinicopathologic and gene expression variables improved the identification of patients with melanoma who may forgo the SLN biopsy procedure because of their low risk of nodal metastasis.

## INTRODUCTION

Primary cutaneous melanoma staging by American Joint Committee on Cancer (AJCC) 8th edition guidelines is determined by whether the disease has spread to sentinel lymph nodes (SLNs).^1,2^ Large multicenter trials have shown that subclinical nodal metastasis is a pivotal prognostic marker^3^ and that SLN biopsy (SLNb) is the standard of care for patients with clinically node-negative melanoma.^4^ The likelihood of SLN metastasis is influenced by tumor thickness quantified as Breslow thickness and other adverse features, such as tumor ulceration and younger age. Rates of nodal metastasis range from 2.5% in very-thin, nonulcerated melanoma (< 0.75 mm Breslow thickness) to 32.9% in thick melanoma (> 3.5 mm Breslow thickness).^3,5,6^

**CONTEXT**

**Key Objective**
There can be uncertainty about whether a sentinel lymph node (SLN) biopsy is warranted in patients with cutaneous melanoma. Aggressive melanoma easily metastasizes, including to SLNs, and positive SLNs identify patients in need of adjuvant therapy. However, most melanomas do not metastasize to SLNs, and the removal of negative SLNs has no discernible therapeutic effect. The key objective of this study was to identify primary melanoma clinicopathologic (CP) variables and a gene expression profile (GEP) that associate with a low risk of SLN metastasis.
**Knowledge Generated**
CP variables in combination with an eight-gene GEP tied to epithelial-to-mesenchymal transition as a biologic process inherent to metastasis effectively stratified melanoma according to its likelihood of SLN metastasis.
**Relevance**
Our CP-GEP model promises to work as an SLN biopsy reduction tool. Patients with negative results may forego SLN biopsy because their risk of nodal metastasis is low.

At present, the only method to accurately determine nodal metastasis is the meticulous pathologic examination of surgically removed SLNs. Per current guidelines (Table 1), SLNb is not recommended if the risk of nodal metastasis is < 5%, as in melanoma with a Breslow thickness of < 0.8 mm and no adverse features. SLNb should be considered if the risk of nodal metastasis is between 5% and 10% (Breslow thickness, 0.8-1.0 mm) and is recommended if the risk of nodal metastasis exceeds 10% (Breslow thickness, > 1.0 mm). Nodal metastasis is found in < 20% of patients who undergo an SLNb.^3^ All patients who undergo SLNb face a > 10% risk of short- and long-term complications, including bleeding, infection, lymphocele, lymphatic fistula, pain, neuropathy, and lymphedema,^7^ as well as an up to 5% risk of hospital readmission within 30 days because of postsurgical complications.^8^ Better methods are needed to identify patients whose risk of nodal metastasis is so low that they may safely forgo SLNb. Here, we report the design of a model that combines established clinicopathologic (CP) variables with a gene expression profile (GEP) to identify patients who have, on average, a risk of nodal metastasis of < 5%. The CP-GEP model may help to identify patients who may forgo SLNb and target the procedure to those most likely to benefit.

**TABLE 1.**
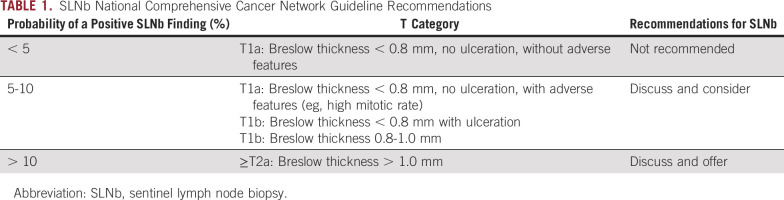
SLNb National Comprehensive Cancer Network Guideline Recommendations

## PATIENTS AND METHODS

### Patient Cohort

Our cohort consisted of 754 patients who had an SLNb performed within 90 days of their diagnosis (ie, a time interval shown to not affect SLNb positivity rates).^9^ Patients with primary cutaneous melanoma who presented at Mayo Clinic tertiary care centers in Minnesota, Arizona, or Florida between 2004 and 2018 with known SLN status were retrospectively identified by electronic searches of pathology reports. Charts were then reviewed for eligibility criteria (see next paragraph), and if met, diagnostic biopsy tissue was requested. Patients underwent SLNb between March 2004 and March 2018. Of the 754 patients in this cohort, 373 were included in a previously published cohort.^10^ All specimens were analyzed by quantitative polymerase chain reaction (PCR) between February 2018 and October 2018.

Eligibility was determined on the basis of histopathology data derived from patient medical records and established by two or more board-certified Mayo Clinic dermatopathologists. Inclusion was determined by the AJCC 7th edition on the basis of institutional practice guidelines of the Mayo Clinic for recommending SLNb, which were based on Breslow thickness, ulceration, mitoses, and age. Patients were eligible for this study if they met one of three conditions, which included Breslow thickness of 1.0-4.0 mm; Breslow thickness of 0.75-0.99 mm and presence of ulceration, mitoses, and/or age < 40 years; or Breslow thickness of 0.50-0.74 mm and presence of at least two of the following: ulceration, mitoses, and age < 40 years. Lesions with a Breslow thickness of > 4 mm were excluded because they were considered a priori high-risk lesions with a rate of nodal involvement > 40%.^3,11,12^ Data analysis was based on the AJCC 8th edition staging system.

**FIG A1.**
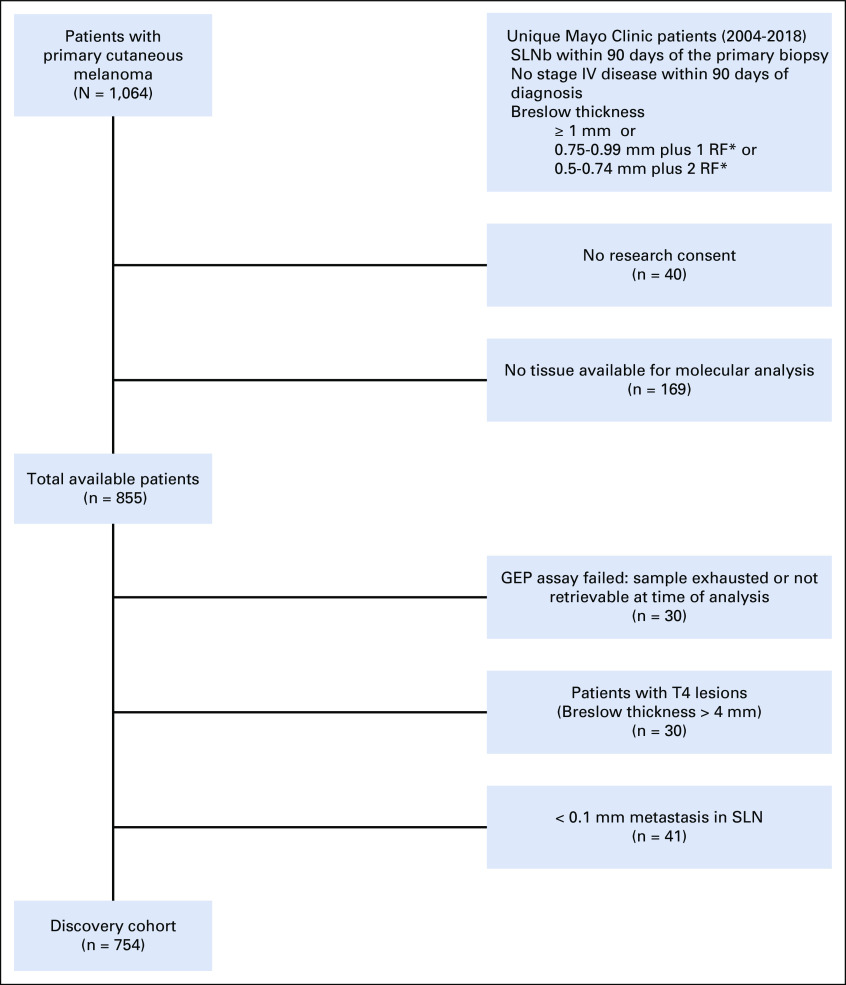
Study flow diagram. The figure depicts the enrollment of patients, inclusion and exclusion criteria, and other potential sources of retrospective bias such as the absence of research consent, the unavailability of tissue for molecular analysis, or failed gene expression profiling (GEP). (*) Risk factors were age < 40 years, mitotic rate > 0/mm^2^, and tumor ulceration present. SLN, sentinel lymph node; SLNb, sentinel lymph node biopsy.

Exclusion criteria were M1 disease within 90 days of primary diagnosis; insufficient primary tumor diagnostic biopsy tissue; inadequate RNA harvested; and, for Minnesota, denial of access to medical records for research purposes (per Minnesota State law). Because there is an ongoing debate about the relevance of < 0.1 mm metastasis in SLN (ie, isolated tumor cells [ITCs] and cell clusters < 0.1 mm in diameter), patients with < 0.1 mm metastasis were excluded from model development. SLNs may harbor clusters of benign melanocytes, particularly in juxtaposition to the capsule. Isolated benign melanocytes and histiocytic melanophages can be present elsewhere in the SLN and mimic ITCs.^13^ Some authors cautioned against hidden tumor burden in < 0.1 mm metastatic SLN and highlighted the need for enhanced pathology assessment protocols.^14,15^ Others found that < 0.1 mm metastasis has no impact on prognosis compared with negative SLNs.^16,17^ Enrollment of patients and inclusion and exclusion criteria are summarized in Appendix Figure A1. Clinical variables used for statistical modeling are listed in Appendix Table A1. This study was approved by the Mayo Clinic institutional review board.

### Gene Expression by Quantitative PCR

See the Appendix for details.

### Statistical Methods

#### Logistic regression and least absolute shrinkage and selection operator.

All classifiers were logistic regression models. Feature selection and parameter estimation were performed through a penalized maximum likelihood estimation algorithm through least absolute shrinkage and selection operator (LASSO).^18^ Models were constructed and analyzed in R 3.4.4 (R Foundation for Statistical Computing, Vienna, Austria) with the package glmnet (version 2.0-16). LASSO was chosen to enhance the interpretability of the model by reducing the number of features while preserving the prediction accuracy. Gene expression input for the regression models was ΔCt. Categorical variables were represented through binary indicator variables. We detected and removed features with a high degree of collinearity using the R package olsrr (version 0.5.1). Features with a tolerance ≤ 0.15 were removed from the input data set (the tolerance represents the fraction of variance in the *k*^th^ feature that cannot be accounted for by other features). The output of logistic regression models estimated the probability of SLN metastasis and was converted into binary results: Samples with a probability of metastasis greater than the cutoff were classified as positive, whereas samples with a probability lower than the cutoff were classified as negative. The performance metrics of the classifiers are listed in Appendix Table A2 and are cutoff specific, except the area under the receiver operating characteristic curve (AUC).

#### Double-loop cross-validation.

**FIG A2.**
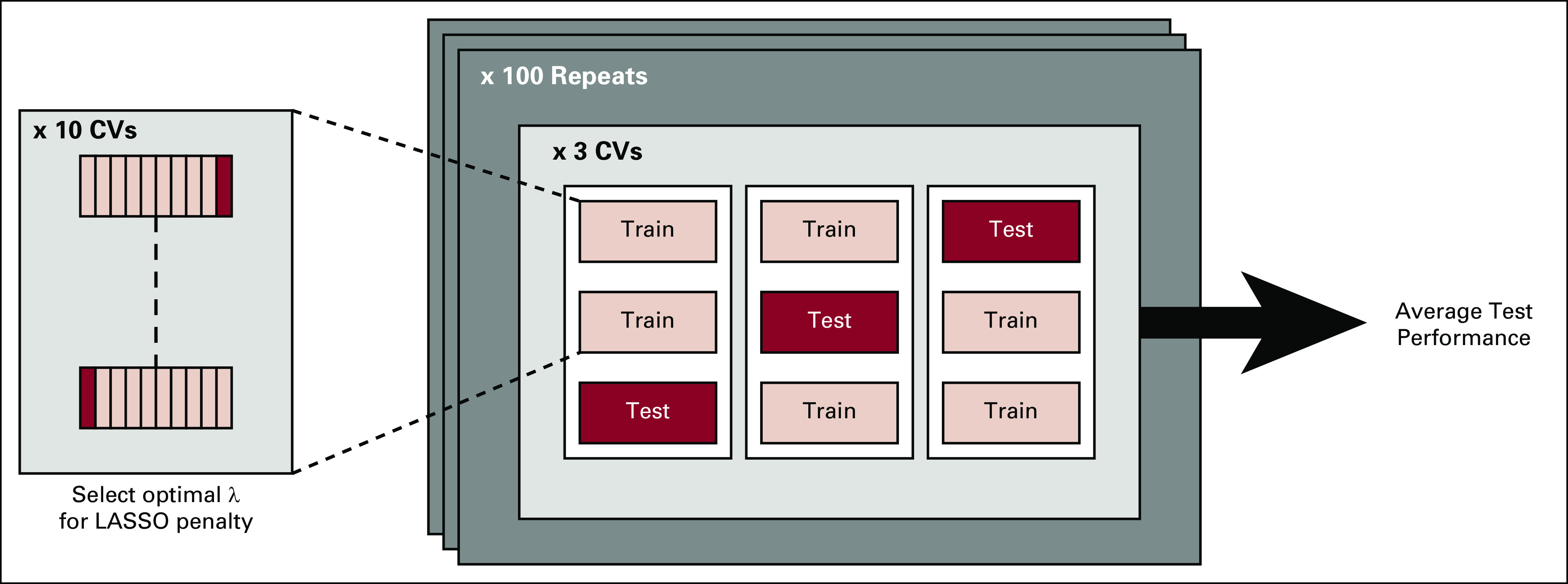
Double-loop cross-validation (DLCV) training-validation protocol in a schematic format. The DLCV consists of two nested cross-validation loops: in the inner loop (tenfold cross-validation), we estimate the optimal λ parameter, namely, the weight of the LASSO penalty term (ie, optimal feature selection); in the outer loop (threefold cross-validation), we assess the performance of the classifier on each test set, with the λ parameter as determined in the training set. Moreover, in each training set of the outer loop, we choose and fix an operating point on the receiver operating characteristic curve, and we assess the performance of the classifier at that operating point in the corresponding test set. The cross-validation procedure has been repeated 100 times, and unless otherwise stated, we reported the average performance over 300 test sets (three test sets per outer loop, repeated 100 times). LASSO, least absolute shrinkage and selection operator.

It is a common requirement in the medical literature that the performance of a new model be validated in a test set independent from the development set. However, splitting the available data just once into a training set and a test set may be viewed as inefficient.^19^ A better solution is to estimate the average performance of the model by repeated cross-validation or bootstrapping. Here, we opted for a repeated cross-validation scheme (ie, double-loop cross-validation [DLCV]).^20^ The key idea of DLCV is to get a reliable estimate of the out-of-sample performance of a classifier by averaging the performance of multiple classifiers trained in cross-validation a number of times (Appendix Fig A2). See the Appendix for details.

#### Memorial Sloan Kettering Cancer Center Nomogram.

See the Appendix for details.

## RESULTS

### Epithelial-to-Mesenchymal Transition in High-Risk Melanoma

To identify candidate genes tied to biologic processes inherent to metastasis and differentially expressed between metastatic and nonmetastatic melanoma, we first reviewed RNA sequencing data obtained previously.^10^ Genes with a false discovery rate of < 0.01 in a comparison of either benign nevi and cutaneous melanoma or cutaneous melanoma with and without SLN metastasis were selected for further qualification. A total of 194 candidate biomarkers and 3 control genes were screened for performance in Breslow thickness and age-matched case-control studies by quantitative PCR (Appendix Table A3). Of the candidate biomarkers, 108 were selected for further analysis in a prospectively designed archival cohort. We noted that genes predictive of nodal metastasis had been associated with epithelial-to-mesenchymal transition (EMT), a biologic process known to promote metastasis in primary cutaneous melanoma.^21^

**FIG A3.**
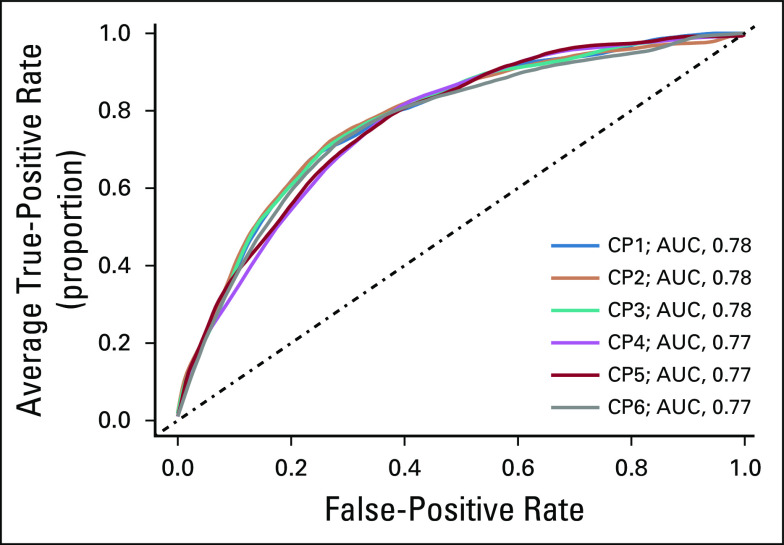
Clinicopathologic (CP) models for predicting sentinel lymph node metastasis. Average receiver operating characteristic curves for six different clinicopathologic models (CP models). CP1: Breslow thickness, age; CP2: Breslow thickness, age, Clark level, ulceration, biopsy location; CP3: Breslow thickness, age, ulceration, mitotic rate as a categorical variable (0, 1 to 6, or > than 6, mitosis per mm^2^); CP4: Breslow thickness, age, ulceration, mitotic rate as a continuous variable; CP5: age mitotic rate, T categories; CP6: Breslow thickness, age, Clark level, mitotic rate, ulceration, angiolymphatic invasion, tumor-infiltrating lymphocytes, biopsy location (as in the MSKCC nomogram), regression, histologic type. AUC, area under the receiver operating characteristic curve; MSKCC, Memorial Sloan Kettering Cancer Center.

Our prospectively designed archival cohort^22^ comprised 754 patients with thin- and intermediate-thickness primary cutaneous melanoma who underwent an SLNb within 90 days of diagnosis (Table 2). Of 754 patients, 128 (17%) were SLN positive, in agreement with the typical prevalence in an SLNb-eligible population.^3^ Our approach was to develop models of the likelihood of SLN metastasis on the basis of either CP variables (CP models) or GEPs of the primary tumor (GEP models) and then to assess the performance of a combined model of CP and GEP factors (CP-GEP models). All models were logistic regression models. Widely available CP factors considered included Breslow thickness, ulceration, mitotic rate, and patient age at diagnosis. Of these, LASSO selected Breslow thickness and patient age. More complex CP models did not improve performance (Appendix Fig A3). We therefore concluded that a CP model that is based on Breslow thickness and patient age is an adequate reference and that there is a limit to the ability of CP factors to predict SLN metastasis.

**TABLE 2.**
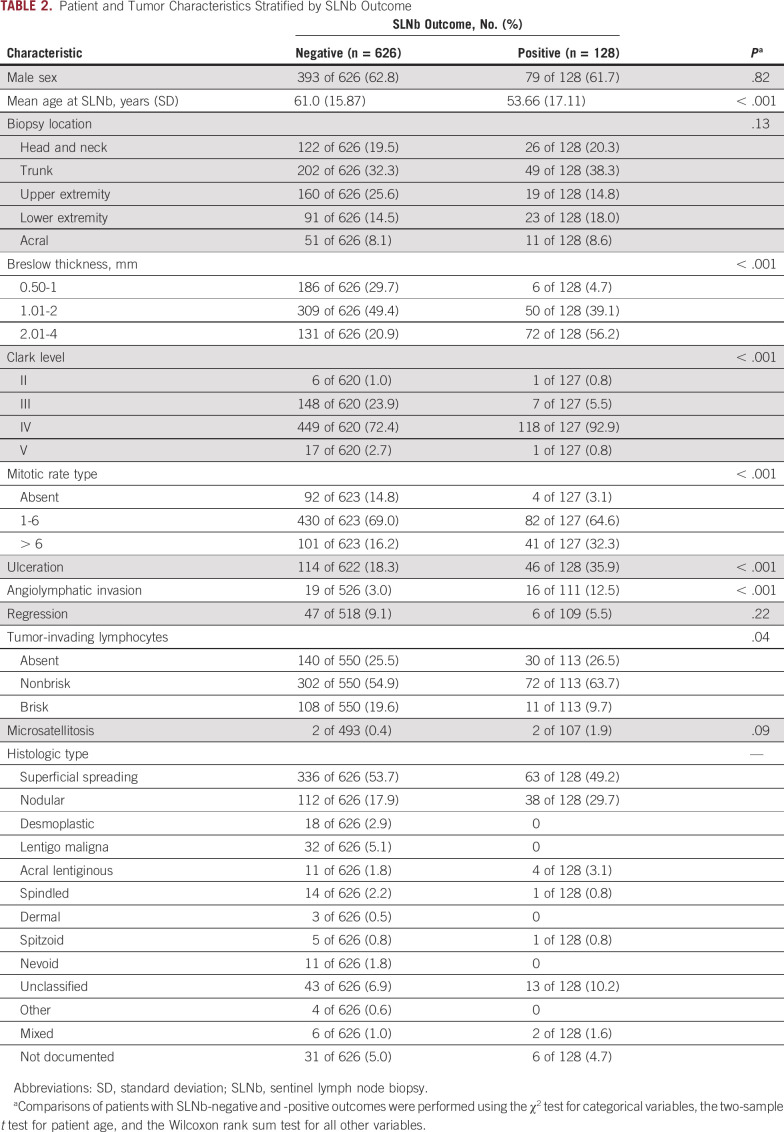
Patient and Tumor Characteristics Stratified by SLNb Outcome

### Using Gene Expression to Predict SLN Metastasis

**FIG 1.**
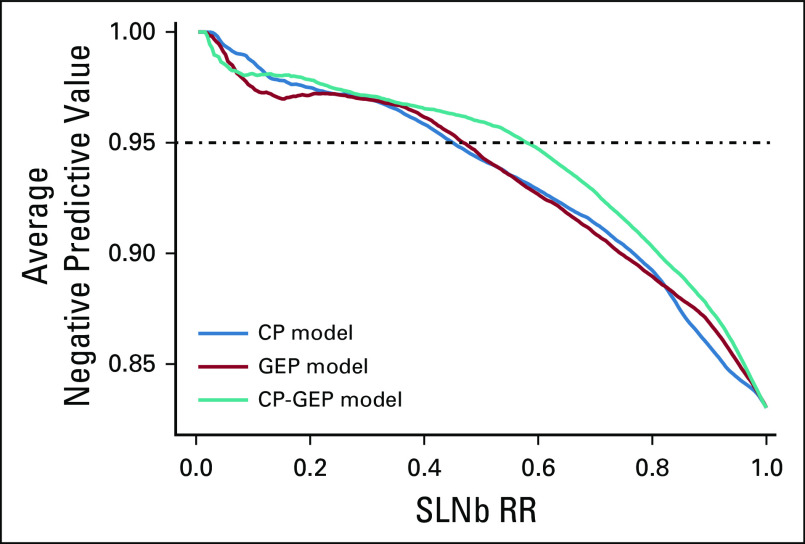
Sentinel lymph node biopsy reduction rates (SLNb RRs) *v* negative predictive value. Shown are the models that are based on clinicopathologic (CP) variables (CP model), gene expression profile (GEP model), and combined GEP and CP variables (CP-GEP model). Curves are averages over 300 double-loop cross-validation–generated test sets.

**FIG A4.**
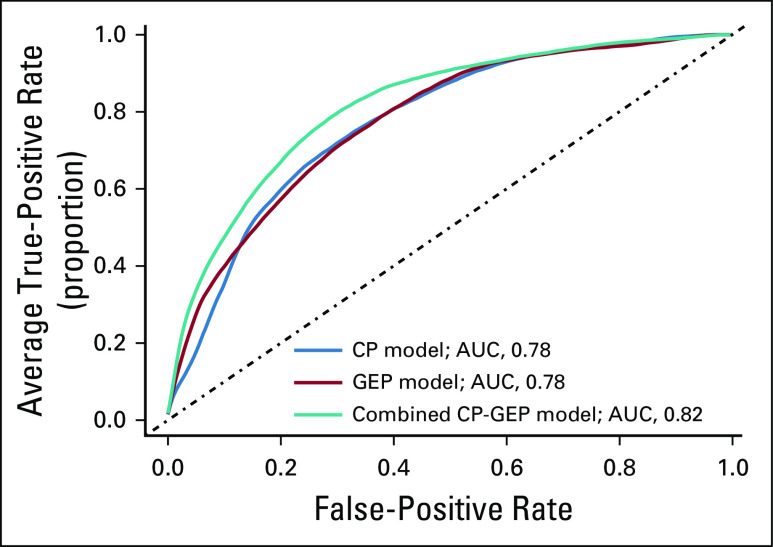
A combined clinicopathologic (CP) and molecular model for predicting sentinel lymph node metastasis. Blue line, model based on CP variables (CP model); red line, model based on gene expression profile (GEP model); teal line, model based on combined GEP and CP variables (CP-GEP model). For each receiver operating characteristic curve, the average area under the curve (AUC) is reported. Curves are averages over 300 double loop cross-validation–generated test sets.

DLCV and LASSO were used to identify a GEP defined from 11 genes that differentiated the patients with and without nodal metastasis detected by SLNb within 90 days of primary diagnosis: ADAM metallopeptidase domain 12 (*ADAM12*), interleukin 8 (*CXCL8*), growth differentiation factor 15 (*GDF15*), integrin-β3 (*ITGB3*), galectin 1 (*LGALS1*), lysyl oxidase like 4 (*LOXL4*), melanoma antigen recognized by T cells 1 (*MLANA*), tissue-type plasminogen activator (*PLAT*), protein kinase C-β (*PRKCB*), glia-derived nexin (*SERPINE2*), and transforming growth factor-β (TGF-β) receptor 1 (*TGFBR1*). Finally, logistic regression modeling was used to develop a novel model combining CP factors (ie, Breslow thickness and patient age) and a GEP. The combined CP-GEP model was based on the expression of *MLANA*, a melanosome marker,^23^ and seven genes functionally linked to EMT and with specific roles in angiogenesis/hypoxia and coagulation: *GDF15*,^24-26^ *CXCL8*,^27,28^ *LOXL4*,^29,30^ *TGFBR1*,^31,32^ *ITGB3*,^33-36^ *PLAT*,^37,38^ and *SERPINE2*^39,40^ (Table 3). The overall discriminatory ability of the CP model (AUC, 0.78; 95% CI, 0.74 to 0.82) and GEP (AUC, 0.78; 95% CI, 0.73 to 0.82) was improved by combining CP factors and a GEP (AUC, 0.82; 95% CI, 0.78 to 0.86; Appendix Fig A4; Appendix Table A4). Likewise, the combined CP-GEP model achieved an approximately 15% higher SLNb reduction rate compared with the benchmark CP model at a negative predictive value of 95% (Fig 1) as well as an almost 60% improvement over current clinical practice as listed in Table 1. The CP-GEP model, therefore, promised to work as an SLNb reduction tool: Patients with a negative test may forgo SLNb because their risk of nodal metastasis is, on average, < 5%, a reduction from the pre-test probability^41^ (Table 1).

**TABLE 3.**
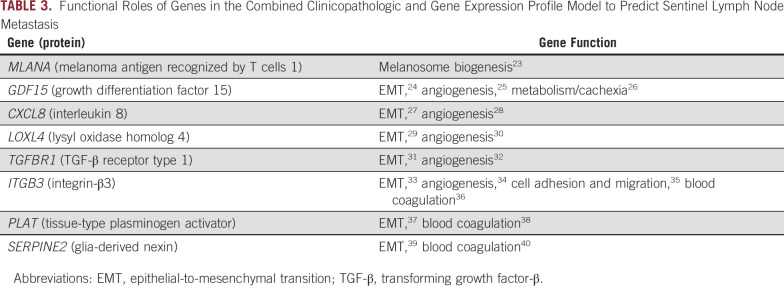
Functional Roles of Genes in the Combined Clinicopathologic and Gene Expression Profile Model to Predict Sentinel Lymph Node Metastasis

For a predictor of SLN status to be clinically relevant, it must change the pretest probability within each T category of melanoma. T categorization provides a valuable risk prediction tool and is readily available in clinical practice. We therefore stratified results of the CP-GEP model by T category. SLNb reduction rates were highest for T1b melanoma at approximately 80% and then decreased as lesions became more advanced (Table 4). T2a melanoma still showed a considerable SLNb reduction rate of 48% while preserving a high negative predictive value of 95%. The high SLNb reduction rate for T1b melanoma is particularly meaningful in light of the increasing incidence of thinner melanoma,^42^ for which CP variables are less predictive.^5^

**TABLE 4.**
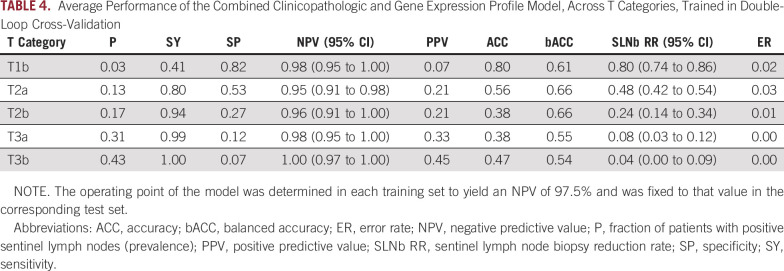
Average Performance of the Combined Clinicopathologic and Gene Expression Profile Model, Across T Categories, Trained in Double-Loop Cross-Validation

**FIG 2.**
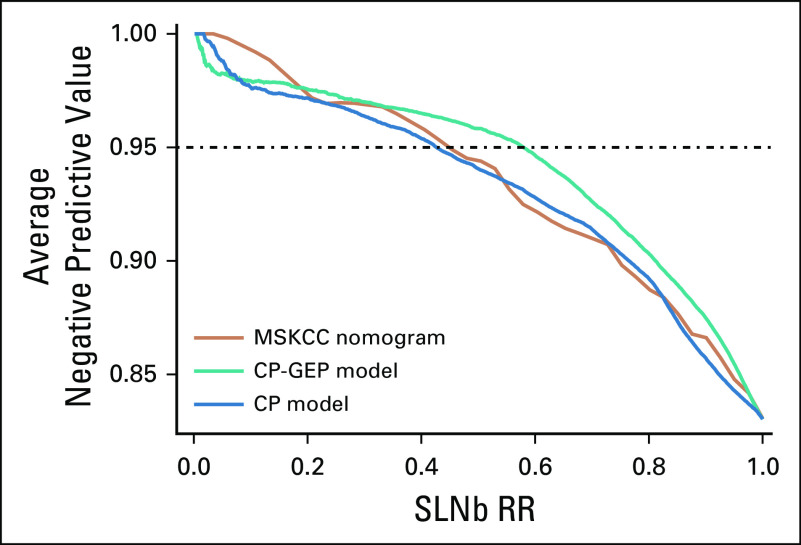
Sentinel lymph node biopsy reduction rates (SLNb RRs) for the Memorial Sloan Kettering Cancer Center (MSKCC) nomogram *v* alternative models. Shown are the models that are based on the MSKCC nomogram, clinicopathologic variables (CP model), and gene expression profile (GEP) and CP variables combined (CP-GEP model). Curves are averages over 100 repeats obtained by concatenating the threefold cross-validation test results.

**FIG A5.**
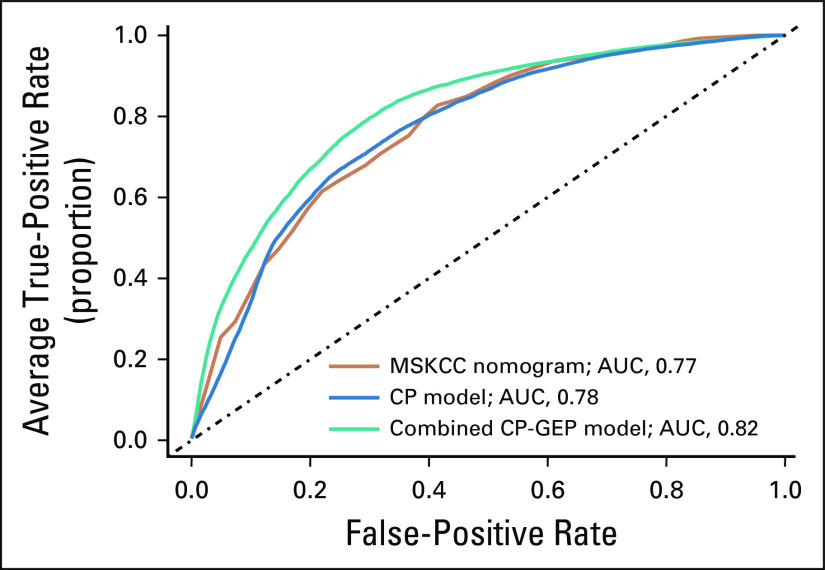
Memorial Sloan Kettering Cancer Center (MSKCC) nomogram performance versus a combined clinicopathologic (CP) and gene expression profile (GEP). Orange line, model based on MSKCC nomogram; blue line, model based on CP variables (CP model); teal line, model based on gene expression and CP variables combined (CP-GEP model). For each receiver operating characteristic curve the average area under the curve (AUC) is reported. Curves are averages over 100 repeats obtained by concatenating the threefold cross-validation test results.

To further define the clinical relevance of the CP-GEP model, we compared CP-GEP performance to the well-known Memorial Sloan Kettering Cancer Center (MSKCC) nomogram for predicting SLN metastasis. The MSKCC nomogram is a graphical representation of a linear predictor developed from a logistic regression model. It is based on five CP variables: patient age, Breslow thickness, Clark level, biopsy location, and tumor ulceration. We found that the MSKCC nomogram performed similarly to the CP model but was outperformed by the CP-GEP model in AUC (Appendix Fig A5) and SLNb reduction rate (Fig 2).

## DISCUSSION

While completion lymphadenectomy for which SLNb was a key determinant has fallen out of favor,^43,44^ SLNb continues to determine patient eligibility for adjuvant therapy. Unfortunately, the majority of SLNb procedures performed today are negative, which confirms only the low-risk nature of the primary tumor without influencing decision making toward adjuvant therapy. Here, we present a model that considers gene expression and CP variables (ie, Breslow thickness and patient age) to assess the likelihood of SLN metastasis in patients diagnosed with thin- and intermediate-thickness primary cutaneous melanoma. The ability to characterize melanoma at the molecular level reduces the need for SLNb, a surgical procedure that carries a risk of complications.^7^ Our approach of combining CP factors with molecular profiling better identifies patients who may forgo the SLNb procedure because of their low risk of metastasis.

For melanoma with a 5%-10% chance of SLN metastasis (Breslow thickness, 0.8-1 mm), SLNb is optional but should be discussed with the patient.^45^ Even though SLNb in this risk group is optional, > 50% of affected patients in the United States undergo SLNb.^46^ The majority of these patients have negative SLNb findings, which highlights our current dilemma with melanoma risk stratification and the limitations of histopathology alone as a predictor of regional metastasis. Multivariable models have used Breslow thickness, tumor ulceration, and patient age to predict SLN status, with age being a negative predictor and Breslow thickness as well as tumor ulceration being strong positive predictors.^47-50^ Angiolymphatic invasion was also found to positively correlate with SLN metastasis in some models.^5,51^ The most ambitious CP models, such as those developed from a large bi-institutional series, achieved SLNb reduction rates of 18%-30%, with a negative predictive value ranging from 93% to 97%.^41^ Attempts at implementing these models into clinical practice have failed because of their limited efficacy. In comparison, the CP plus molecular model developed here showed an SLNb reduction rate of 42% at a negative predictive value of 96% (Appendix Table A4). LASSO applied to CP factors in this cohort identified Breslow thickness and patient age as sufficient for CP risk stratification. More complex CP models did not improve performance (Appendix Fig A3). There seems to be a clear limit in the ability of CP factors to predict SLN metastasis.

To improve the performance of predictive models, we developed a GEP from primary diagnostic biopsy tissue. GEP has been used successfully in breast cancer to individualize therapy.^52^ Previous research on gene expression in invasive breast cancer,^53^ prostate cancer,^54^ colon cancer,^55^ melanoma,^10^ and other solid cancers^56^ has consistently demonstrated the upregulation of adhesion receptors and secreted factors that remodel the tumor microenvironment and are involved in EMT.^10,53-57^ Here, we have confirmed this upregulation and found genes involved in EMT with specific roles in angiogenesis (growth differentiation factor 15,^25^ interleukin 8,^28^ lysyl oxidase homolog 4,^58^ TGF-β receptor type 1,^32^ and integrin β3^34^) and coagulation (tissue-type plasminogen activator,^38^ and glia-derived nexin^40^) as well as the melanosome biogenesis marker melanoma antigen recognized by T cells 1^23^ to be associated with SLN metastasis (Table 3). The functional roles of these genes have been demonstrated by genetic approaches^32,34,38,59^ and pharmacologic efficacy studies where the inhibition of integrin-β3 by cyclic peptide,^60^ TGF-β receptor type 1 by kinase inhibitor,^61^ and interleukin 8 by neutralizing antibody^62^ reduced tumor angiogenesis, tumor growth, and metastasis. Tumor vascularity in melanoma diagnostic biopsy tissue is well known to associate with nodal and distant metastasis but has been difficult to quantify in the past.^63^ Likewise, constitutive fibrinolytic activity in tumor tissue has been described as early as 1911^64^ and attributed largely to plasminogen activators^38^ and other serine proteases, such as glia-derived nexin,^65^ which promote metastasis,^66^ disseminated intravascular coagulation, and bleeding in patients with metastatic cancer.^67^

A drawback of the simultaneous selection of CP variables and genes by our feature selection algorithm is the absence of established variables easily recognizable by clinicians, such as ulceration, in the CP-GEP model. Our retrospective study was also limited by referral bias and variations in pathologic assessment. The exclusion of patients with ambiguous < 0.1 mm metastasis from model development could have influenced the results. Moreover, we excluded T4 lesions (ie, melanoma with a Breslow thickness < 4 mm) because the pretest probability of regional metastasis for these patients is very high. For example, 21 (70%) of 30 patients with T4 lesions in our cohort presented with regional metastasis, which is well above the recommend threshold for recommending SLNb. Clinicians may not want to forgo SLNb for an a priori high-risk T4 melanoma, even if a molecular classifier was available. Finally, eligibility of patients with T1 melanoma was determined by the Mayo Clinic institutional practice guidelines for recommending SLNb, which select for higher-risk patients, such as those < 40 years of age and with T1b melanoma. Indiscriminate inclusion of low-risk patients could have biased test performance calculations. For example, Vetto et al^68^ repurposed a molecular prognostic test to guide SLNb decision making in T1-T2 melanomas: Patients with a negative test would forgo SLNb in virtue of a nodal metastasis risk < 5% (negative predictive value > 95%). However, SLNB to SLNb metastasis risk was only < 5% if cohorts were enriched for a priori low-risk cases, such as by including T1a melanoma or restricting the analysis to patients > 65 years of age.

In summary, CP variables in combination with an eight-gene GEP tied to EMT as a biologic process inherent to metastasis effectively stratified patients with melanoma according to their likelihood of SLN metastasis. Previous attempts to develop molecular risk factors have been limited by small cohort sizes, incomplete TNM staging data, or limited clinical utility of the resulting models.^49,69,70^ Our approach of combining CP factors and gene expression variables improved the performance of CP factors alone by outperforming current clinical practice and important benchmarks, such as the MSKCC nomogram. The combined CP-GEP model maintained an average negative predictive value of > 95% across pathologic tumor thickness categories, which highlights its promise as a tool to identify low-risk patients who may forgo SLNb. Our findings are particularly relevant to patients with thin- and intermediate-thickness melanoma who demonstrate significant heterogeneity in their SLN metastasis risk.^2^ Additional research is ongoing to externally validate our results.

## Figures and Tables

**TABLE A1.**
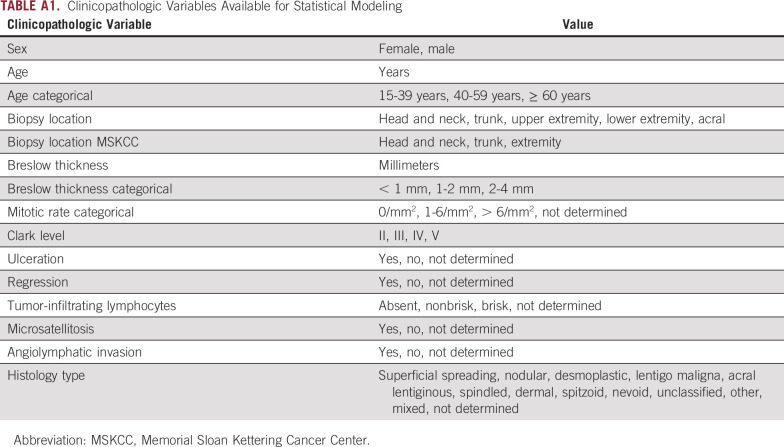
Clinicopathologic Variables Available for Statistical Modeling

**TABLE A2.**
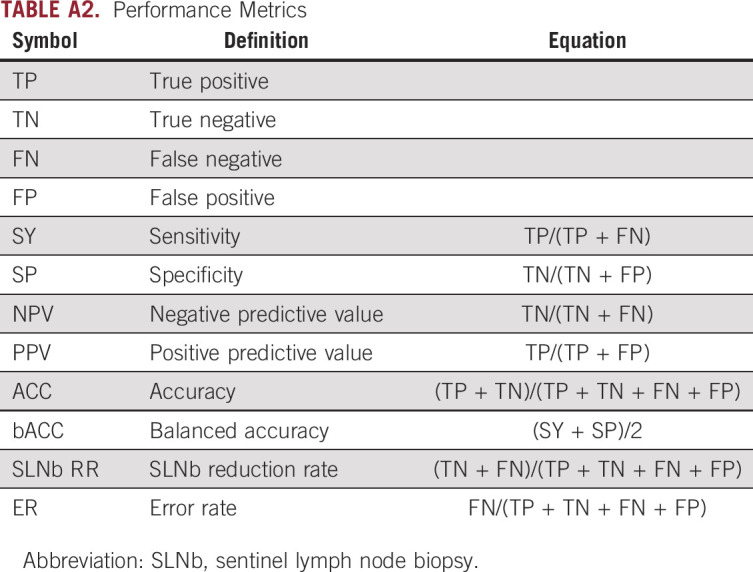
Performance Metrics

**TABLE A3.**
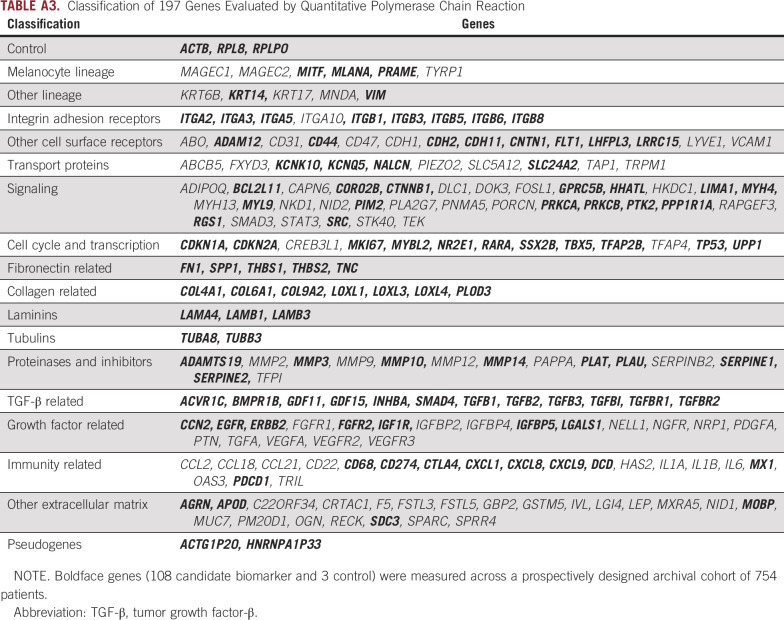
Classification of 197 Genes Evaluated by Quantitative Polymerase Chain Reaction

**TABLE A4.**
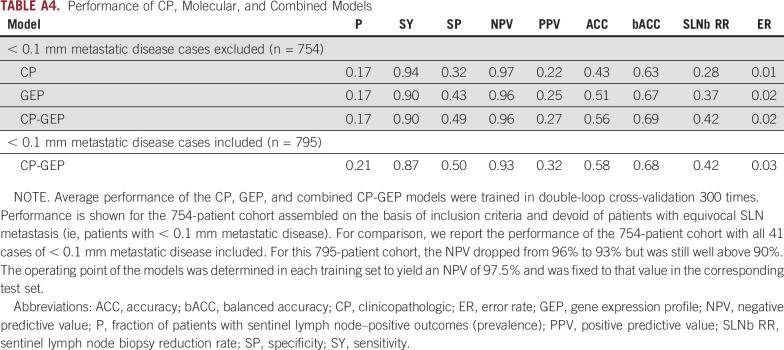
Performance of CP, Molecular, and Combined Models

## APPENDIX

### Methods

Quantitative polymerase chain reaction (PCR) was performed as previously described.^10^ RNA purification was from formalin-fixed paraffin-embedded tissue (QIAGEN, Hilden, Germany). Quantitative reverse transcription PCR was done using the BioMark HD System and dynamic array integrated fluid circuits (Fluidigm, South San Francisco, CA). All cDNA was pre-amplified (TaqMan PreAmp Master Mix, Applied Biosystems, Foster City, CA). Array-based quantitative PCR was with the help of the TaqMan Gene Expression Master Mix (Applied Biosystems). After thermal cycling, raw Ct data were checked for linear amplification. Gene expression was corrected by the mean of housekeeping genes (*RLP0*, *RLP8*, and β-actin) using the ΔCt method.

### Statistical Methods

#### Double-loop cross-validation.

In the double-loop cross-validation scheme, there are two nested cross-validation loops. In the inner loop (10-fold cross-validation), we optimized the λ parameter by determining the number of features (ie, the weight of the least absolute shrinkage and selection operator penalty term), and in the outer loop (threefold cross-validation), we designed a classifier on the training set (two of the three folds). Next, we assessed the performance of the trained classifier on the remaining fold (test set), with the λ parameter fixed to the value estimated in the training set. The operating point of the classifier, as determined by a cutoff value on the estimated probability, was fixed to the training value as well. (We chose an operating point that yielded a negative predictive value of 97.5% in our cohort.) The cross-validation procedure was repeated 100 times. Unless otherwise stated, we report the average performance of 300 test sets (three test sets for each of the 100 repeats). The final classifiers were trained on the entire data set using the average 𝜆 parameter over 300 runs.

#### Memorial Sloan Kettering Cancer Center nomogram.

The majority of online tools for melanoma provide prognostic information^47^ (Zabor et al: Ann Surg Oncol 25:2172-2177, 2018). The Memorial Sloan Kettering Cancer Center (MSKCC) nomogram, in contrast, is a tool specifically designed to predict the probability of primary cutaneous melanoma metastasis to SLN.^23^ The nomogram corresponds to a logistic regression model that is based on five clinicopathologic variables: age (range, 20-95 years), Breslow thickness (range, 0.1-10 mm), Clark level (II, III, IV, or V), biopsy location (trunk, extremity, or head and neck), and tumor ulceration (yes or no). The nomogram is accessible online (MSKCC: https://www.mskcc.org/nomograms/melanoma/sentinel_lymph_node_metastasis). While attempting to apply the nomogram to our cohort, we could not calculate the probability of SLN metastasis for 16 patients because of missing values or because the values were outside the allowable range for the nomogram. Six patients were < 20 years of age, seven had a missing Clark level (one of whom was also < 20 years of age), and four did not have ulceration status available. Therefore, the analysis shown is based on 738 patients.

#### CIs.

CIs for the area under the receiver operating characteristic curve were determined by the R package cvAUC (version 1.1.0); for the other metrics, they were determined by normal approximation interval when applicable or cross-validation estimates otherwise.
